# Literary Notes

**Published:** 1899-11

**Authors:** 


### LITERARY NOTES.

THE United States Marine Hospital Service has been engaged in the investigation of the cause of yellow fever. The Surgeon-General of that branch of the public service has lately sent out a number of brochures bearing upon the study of this disease. The following are their titles:

Report of the commission of medical officers detailed by authority of the President to investigate the cause of yellow fever.

Yellow fever: its nature, diagnosis, treatment and prophylaxis, and quarantine regulations relating thereto.

Train inspection in yellow fever epidemics.

Shipment of merchandise from a town infected with yellow fever.

Other pamphlets recently sent out by the Surgeon-General, U. S. M. H. S. are:

Mortality statistics in the United States for the year ending December 31, 1897.

Report on Formaldehyde disinfection in a vacuum chamber.

Any or all of these papers may be obtained upon application to Surgeon-General Walter Wyman, U. S. M. H. S., Washington, D. C.

THE *Vermont Medical Monthly*, published at Burlington, issued a souvenir number in September, 1899, complimentary to the Vermont State Medical Society. It contained, among other interesting material, a history of the Medical Department of the University of Vermont, historical sketches of the several hospitals at Burlington, the Vermont laboratory of hygiene, and a historical résumé of the City of Burlington. These articles were copiously illustrated with half-tone engravings, and the monthly was printed on highly finished book paper. It is a most creditable exhibit of enterprise on the part of the editors and publishers of this excellent journal.

